# Liver function estimation using multiphase hepatic CT: diagnostic performance of iodine-uptake and volumetric parameters

**DOI:** 10.1007/s00330-025-11497-1

**Published:** 2025-03-13

**Authors:** Yasunori Nagayama, Masamichi Hokamura, Narumi Taguchi, Yasuhiro Yokota, Takumi Osaki, Koji Ogasawara, Shinya Shiraishi, Ryuya Yoshida, Ryota Harai, Masafumi Kidoh, Seitaro Oda, Takeshi Nakaura, Toshinori Hirai

**Affiliations:** Department of Diagnostic Radiology, Graduate School of Medical Sciences, Kumamoto University, Chuo-ku, Japan

**Keywords:** Liver dysfunction, Liver function tests, Multidetector computed tomography, Iodine

## Abstract

**Objectives:**

To investigate whether multiphase hepatic CT can predict liver function measured with indocyanine-green-retention test (ICG-R15) and identify patients with severe liver dysfunction contraindicating major hepatectomy, defined as ICG-R15 ≥ 20%, compared to technetium-^99m^-galactosyl serum albumin (^99m^Tc-GSA) scintigraphy.

**Materials and methods:**

This retrospective study included 118 patients (84 men, mean age, 69.4 ± 11.3 years) who underwent ICG-R15, ^99m^Tc-GSA, and multi-phase CT including early portal-venous-phase and 3-min delayed-phase. CT-derived extracellular volume fraction (ECV), iodine washout rate (IWR), liver and spleen volumes normalized by body-surface-area (LV/BSA and SpV/BSA, respectively), and ^99m^Tc-GSA-derived blood clearance index (HH15) and liver receptor index (LHL15) were quantified. Each parameter was compared between ICG-R15 ≥ 20% (*n* = 22) and ICG-R15 < 20% (*n* = 96) groups. Correlations with ICG-R15 were analyzed. The diagnostic performance to predict ICG-R15 ≥ 20% was assessed with areas under the receiver operating characteristic curve (AUC). Multivariable logistic regression analysis was used to identify independent CT predictors, and combined performance was determined.

**Results:**

In the ICG-R15 ≥ 20% group, IWR (*p* < 0.001), LV/BSA (*p* = 0.026), LHL15 (*p* < 0.001) were lower and ECV (*p* = 0.001), SpV/BSA (*p* = 0.005), and HH15 (*p* < 0.001) were higher compared to ICG-R15 < 20% group. ICG-R15 showed positive correlations with ECV (*r* = 0.355), SpV/BSA (*r* = 0.248), and HH15 (*r* = 0.385), while negative correlations with IWR (*r* = −0.523), LV/BSA (*r* = −0.123, not statistically significant), and LHL15 (*r* = −0.504). The AUC of ECV, IWR, LV/BSA, SpV/BSA, HH15, and LHL15 were 0.719, 0.845, 0.653, 0.694, 0.844, and 0.878, respectively. IWR, SpV/BSA, and LV/BSA were independent predictors, with a combined AUC of 0.924.

**Conclusion:**

IWR predicted liver function better than ECV and hepatosplenic volumetry. The combined IWR and volumetry yielded an accurate prediction of severe liver dysfunction.

**Key Points:**

***Question*** *Despite the widespread use of multiphase CT in patients with hepatobiliary diseases, its potential role in assessing liver function has been scarcely evaluated*.

***Findings*** *Iodine washout rate (IWR), liver volume indexed by body surface area, and spleen volume indexed by body surface area were independent predictors for severe liver dysfunction*.

***Clinical relevance*** *Combined IWR and hepatosplenic volumetry on routine hepatic CT may help assess hepatic function for optimizing treatment strategies and predicting patient prognosis*.

**Graphical Abstract:**

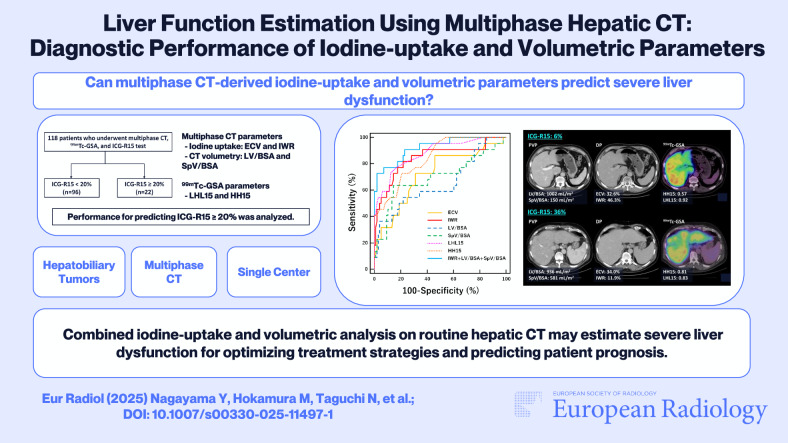

## Introduction

Accurate estimation of liver function is essential for assessing disease severity, monitoring progression, and predicting the prognosis of chronic liver disease. It also facilitates optimal surgical planning to minimize the risk of liver failure, a major cause of mortality and morbidity after hepatectomy [1, 2]. The liver functional assessment is crucial not only in patients with hepatocellular carcinoma (HCC), most of whom have underlying cirrhosis, but also in patients with hepatic metastases due to the rising prevalence of nonalcoholic steatohepatitis and neoadjuvant chemotherapy-induced liver damage [3, 4]. While biochemical parameters and clinical scoring systems such as the Child–Pugh score are commonly used as static liver functional makers, these methods may have limited predictive value in predicting the risk of hepatic decompensation and patient prognosis [5–9].

The indocyanine green (ICG) retention after 15-min (ICG-R15) is a well-established dynamic quantitative test for estimating liver function and determining the safe extent of hepatectomy [10, 11]. The utility of the ICG test has been validated and recognized in European countries [12–14], with the recently developed European guidelines recommending it prior to hepatectomy as a next-step test following static biochemical function assessments [15]. It also has the potential to predict the severity of portal hypertension, the prognosis of cirrhosis, and clinical outcomes following therapeutic interventions other than hepatectomy [12, 16–19]. Specifically, an ICG-R15 ≥ 20% indicates severe liver dysfunction which is considered a contraindication for major hepatectomy [20, 21]. Although the ICG test is used as one of the reference standards for liver function [13, 14, 22–28], it is relatively time-consuming and lacks imaging guidance and morphological information. In contrast, technetium-99m diethylenetriamine-pentaacetic acid galactosyl human serum albumin (^99m^Tc-GSA) scintigraphy is an imaging-based liver function test [29–31]. ^99m^Tc-GSA binds exclusively to the asialoglycoprotein receptor on hepatocytes without being taken up by other organs. Given that decreased receptor expression is indicative of liver damage, ^99m^Tc-GSA scintigraphy provides a direct evaluation of regional hepatocyte function. Consequently, its use is recommended for patients with a limited future liver remnant, underlying liver disease, or when volume modulation strategies are implemented before major hepatectomy [15]. However, there are drawbacks such as limited availability, particularly in Western countries, high cost, technical complexity, and increased radiation exposure, which may limit its broad clinical applicability and frequent use in screening.

Multiphase hepatic CT is widely used for the diagnosis and management of liver tumors. In addition to tumor assessments, CT volumetry can provide valuable quantitative information on the severity of liver fibrosis and portal hypertension [32, 33]. Despite the significant correlations between volumetric indices and hepatic function [13, 28, 34, 35], several studies have shown their limited capability in predicting liver dysfunction and post-operative outcomes due to a lack of information on parenchymal damage [35–37]. As alternative CT approaches, iodine-uptake parameters such as extracellular volume fraction (ECV) and iodine washout rate (IWR) have been proposed as potential liver fibrosis markers [38–41]. Hepatic ECV, which increases in a fibrotic liver due to the accumulation of collagen and other extracellular matrix proteins, can be estimated using equilibrium phase CT [38–40]. The hepatic IWR reflects altered intrahepatic hemodynamics in a fibrotic liver, characterized by reduced hepatic enhancement during the portal venous phase (PVP) and prolonged iodine retention during the delayed phase (DP) [41]. These iodine-uptake parameters may facilitate the assessment of liver function on routine multiphase hepatic CT; however, no studies have yet validated their feasibility in comparison to ^99m^Tc-GSA and CT volumetric indices.

The purpose of this study was to investigate whether multiphase hepatic CT parameters (ECV, IWR, and hepatosplenic volume indices) correlate with liver function measured with ICG-R15 and allow the identification of patients with severe liver dysfunction compared to ^99m^Tc-GSA scintigraphy.

## Materials and methods

This retrospective study was approved by the institutional review board, and the requirement for patient-informed consent was waived.

### Patients

By searching the radiological database at the study institution, patients with hepatobiliary tumors who underwent multiphase hepatic CT and ^99m^Tc‑GSA scintigraphy using a combined single-photon emission CT (SPECT) with multidetector CT (SPECT-CT) system between January 2017 and December 2021 were retrospectively identified. Exclusion criteria comprised: (a) lack of available ICG-R15 data, (b) absence of available laboratory data (including hematocrit [Hct], alanine aminotransferase [AST], aspartate aminotransferase [ALT], platelet count [Plt], prothrombin time international normalized ratio [PT-INR], total bilirubin [T-bil], albumin [Alb], creatinine [Cr]) within one week of CT, (c) patients with portal-venous occlusion, (d) patients after splenic artery embolization, (e) those after hepatectomy or splenectomy, (f) those after transarterial chemoembolization within 1 year of CT, (g) biliary obstruction, or (h) those with diffuse or huge hepatic lesions impeding parenchymal evaluation. Among patients who underwent hepatectomy, post-hepatectomy liver failure (PHLF) was assessed using the International Study Group for Liver Surgery definition [42]. In this study, clinically significant PHLF was defined as increased PT-INR and hyperbilirubinemia requiring treatment deviating from standard clinical management on or after postoperative day 5 (grade B or C) [42]. PHLF grade A, defined as increased PT-INR and hyperbilirubinemia without requiring treatment, was not considered clinically significant PHLF, as no clinical impact for this category has been reported [43, 44]. Perioperative mortality was defined as any death occurring during the same hospital stay or within 3 months after hepatectomy.

### ICG retention test

ICG retention test was conducted by administering an ICG solution (Diagnogreen, Daiichi Pharmaceutical) intravenously at a dose of 0.5 mg/kg. The blood ICG concentration was monitored transcutaneously and the values of ICG-R15 were calculated using a Pulse Dye Densito-Graph Analyzer (DDG-3300K, Nihon Kohden). The results of the ICG test were employed as a reference standard for liver function, similar to previous studies on imaging-based liver function assessments [13, 14, 22–28].

### Biochemical test

As biochemical liver functional markers, the Model for End-stage Liver Disease (MELD) scores were calculated with the following formula:

$${{{\rm{MELD}}}}\; {{{\rm{score}}}}\, = 	 \, 9.57\times {{\mathrm{ln}}}({{{\rm{Cr}}}}[{{{\rm{mg}}}}/{{{\rm{dL}}}}])+3.78\times {{\mathrm{ln}}}({{{\rm{T}}}}-{{{\rm{bil}}}}[{{{\rm{mg}}}}/{{{\rm{dL}}}}]) \\ 	 +11.2\times {{\mathrm{ln}}}({{{\rm{INR}}}})+6.43$$

### Multiphase CT and ^99m^Tc-GSA scintigraphy protocols

CT and ^99m^Tc-GSA images were acquired using a SPECT/CT fusion system that integrates a gantry-free SPECT with dual-head detectors (Tandem Discovery 670, GE) and a multi-detector CT scanner (BrightSpeed, GE). The two systems were positioned adjacent to each other, allowing the CT table to be moved directly into the SPECT scanner. This arrangement facilitates the acquisition of both scintigraphic and CT images without the need to reposition the patient on the same table, enabling the creation of accurately fused images with minimal misregistration [30].

For multiphase CT, 600 mgL/kg of iodinated contrast medium was intravenously administered over 30 s after pre-contrast CT scanning. The scanning delay times were determined using the bolus tracking technique. The arterial phase was scanned 12 s after the abdominal aorta reached 150 HU (approximately 30 s after the start of contrast administration), the early PVP was scanned 15 s after the end of the arterial phase scanning (approximately 50 s after the start of contrast administration), and the delayed phase was scanned 180 s after the start of contrast administration. The scanning parameters for all enhancement phases were a tube voltage of 120 kVp, a tube current of 250 mA, a rotation time of 0.8 s, a beam collimation of 1.25 mm, and a beam pitch of 1.37. All image data were reconstructed using a standard soft kernel with a slice thickness and increment of 5 mm each.

For the scintigraphy imaging, a fixed 185 MBq dose of ^99m^Tc-GSA (Nihon Medi-Physics Co.) was injected as a bolus via the antecubital vein for all patients regardless of body weight according to the prescribing information. Dynamic images were recorded, and time-activity curves for the liver and heart were generated by placing regions of interest (ROIs) on both organs. The blood clearance index (HH15) was calculated by dividing the total heart ROI counts at 15 min by those at 3 min post ^99m^Tc-GSA injection. The liver receptor index (LHL15) was determined by dividing the total liver ROI counts by the sum of the total counts in the ROIs for both the liver and heart at 15 min post-injection. As liver functional reserve decreases, LHL15 decreases, and HH15 increases [45].

### CT Image analysis

A radiologist (Y.Y.) with 8 years of experience in abdominal CT performed blinded image analyses without knowledge of patient information. All quantifications were also performed by a different radiologist (N.T.) with 12 years of experience in abdominal CT to assess the inter-reader agreement. With pre-contrast, PVP, and DP images, circular region-of-interests (ROIs) were placed on anterior, posterior, medial, and lateral sections of the liver. Each ROI was approximately 250 mm^2^, carefully avoiding apparent intrahepatic vessels, bile ducts, lesions, and artifacts. The mean hepatic attenuation (HU) at each phase was calculated from four ROIs. On pre-contrast and DP images, the attenuation of the aorta at the mid-hepatic level was measured for ECV estimation. ROIs were first placed on the DP images, then copy-pasted on the pre-contrast and PVP images with appropriate placement correction if needed. The contrast enhancement of each object was measured by subtracting pre-contrast from contrast-enhanced attenuation. The ECV was calculated as [38–40]:

$${{{\rm{ECV}}}}=\frac{{{{\rm{Hepatic}}}}\; {{{\rm{enhancement}}}}\; {{{\rm{during}}}}\; {{{\rm{DP}}}}\times (100-{{{\rm{Hct}}}}\, \left[ \% \right])}{{{{\rm{Aortic}}}}\; {{{\rm{enhancement}}}}\; {{{\rm{during}}}}\; {{{\rm{DP}}}}}$$

The IWR was calculated using the following equations [41]:

$${{{\rm{IWR}}}}= 	 \frac{{{{\rm{Hepatic}}}}\; {{{\rm{enhancement}}}}\; {{{\rm{during}}}}\; {{{\rm{PVP}}}}-{{{\rm{Hepatic}}}}\; {{{\rm{enhancement}}}}\; {{{\rm{during}}}}\; {{{\rm{DP}}}}}{{{{\rm{Hepatic}}}}\; {{{\rm{enhancement}}}}\; {{{\rm{during}}}}\; {{{\rm{PVP}}}}} \\ 	 \times 100$$

CT volumetric analysis was conducted to measure liver volume (LV) and splenic volume (SpV) with the PVP images using an automated segmentation software (SYNAPSE VINCENT®, Fujifilm Medical). The tumor volume was excluded from LV measurement. To account for differences in body size, LV and SpV were normalized by the body surface area (BSA) calculated using the following formula: √[body weight (kg) × height (cm)/3600] [46].

### Statistical analysis

Statistical analyses were performed using MedCalc software. Continuous variables are presented as mean ± standard deviation after confirming normality with the Kolmogorov–Smirnov test, and categorical variables are presented as numbers and percentages. Spearman rank correlations were used to assess the association of ICG-R15 with CT parameters (ECV, IWR, LV/BSA, and SpV/BSA), and ^99m^Tc-GSA (HH15 and LHL15). Welch tests or Fisher’s exact tests were used to comparing clinical data and imaging parameters between binarized patient groups (ICG < 20% vs ICG ≥ 20%). Receiver operating characteristic (ROC) analysis was performed to assess the diagnostic performance to predict contraindications to major hepatectomy (ICG ≥ 20%). Sensitivity and specificity with optimal cutoff values were determined using the Youden index. The DeLong method was used to compare the area under the ROC curves (AUC) between each CT parameter and ^99m^Tc-GSA. Multivariable logistic regression analyses were conducted to identify independent CT parameters for predicting ICG-R15 > 20%; the AUCs of the model combining independent predictors were determined. For iodine-uptake parameters, inter-observer reproducibility was assessed with intraclass-correlation-coefficients (ICC). A *p*-value < 0.05 was considered statistically significant.

## Results

### Patient demographics

A total of 331 patients met the inclusion criteria. Among these, the following were excluded: 54 due to lack of ICG-R15 data, 29 for missing laboratory data, 28 with portal venous occlusion, 4 following splenic artery embolization, 24 post-hepatectomy, 7 post-splenectomy, 8 post-transarterial chemoembolization, 19 with biliary obstruction, and 20 with multiple or large hepatic tumors. Consequently, the final sample consisted of 118 patients (84 men, mean age of 69.4 ± 11.3 years) (Table 1). Among these, 72 patients (52 men, mean age 68.9 ± 10.9 years) underwent hepatectomy, with no major hepatectomies performed on those with severe liver dysfunction (ICG-R15 ≥ 20%). PHLF requiring treatment occurred in two patients (2.8%), and there was no perioperative mortality.

**Table 1:** Patient demographics

Parameter	Value
Age (years)	69.4 ± 11.3
Male:female	84 (71.1):34 (28.9)
Body mass index (kg/m^2^)	23.8 ± 3.8
Body surface area (m^2^)	1.67 ± 0.2
Etiologies	
Hepatitis C virus	30 (25.4)
Hepatitis B virus	15 (12.7)
Non-B non-C hepatitis	9 (7.6)
Alcohol	18 (15.2)
Nonalcoholic steatohepatitis	13 (11.0)
Autoimmune liver disease	1 (0.8)
No chronic liver disease	32 (27.1)
Fibrosis stages*	
F0/F1/F2/F3/F4	8 (11.1)/20 (27.8)/11(15.3)/18 (25.0)/15 (20.8)
Child–Pugh classes	
A/B/C	111 (94.0)/6 (5.1)/1 (0.9)
Biochemical data	
Platelet count (10^9^/L)	174.4 ± 60.6
ALT (U/L)	33.9 ± 18.6
AST (U/L)	31.3 ± 21.8
Total bilirubin (mg/dL)	0.9 ± 0.5
PT-INR	1.1 ± 0.2
MELD score	4.03 ± 4.47
ICG-R15 (%)	14.2 ± 11.2

Data are mean ± standard deviation or number (%)

*ALT* alanine aminotransferase, *AST* aspartate aminotransferase, *PT-INR* prothrombin time-international normalized ratio, *MELD* model for end-stage liver disease, *ICG-R15* indocyanine green retention after 15 min

^*^ Pathological liver fibrosis data was available in 72 patients

### Comparisons of imaging parameters according to ICG-R15

Patients with ICG-R15 ≥ 20% showed significantly lower hepatic enhancement during PVP, higher ECV, lower IWR, lower LV/BSA, higher SpV/BSA, higher HH15, and lower LHL15 than patients with ICG-R15 < 20% (all *p* ≤ 0.026, Table 2). The ICC for hepatic enhancement during PVP and DP, ECV, and IWR were 0.974 (95% CI: 0.962–0.982), 0.947 (95% CI: 0.924–0.963), 0.882 (95% CI: 0.829–0.918), and 0.969 (95% CI: 0.956–0.979), respectively, indicating excellent inter-reader reproducibility.

**Table 2:** Comparisons of parameters between patients with ICG-R15 ≥ 20% and < 20%

Parameters	ICG-R15 < 20% (*n* = 96)	ICG-R15 ≥ 20% (*n* = 22)	*p*-value
Age (years)	68.4 ± 11.4	73.9 ± 10.1	0.036
Male/female	66 (68.8)/30 (31.2)	18 (81.8)/4 (18.2)	0.300
Body mass index (kg/m^2^)	23.6 ± 4.0	25.0 ± 2.8	0.052
Body surface area (m^2^)	1.67 ± 0.21	1.71 ± 0.18	0.428
Iodine parameters			
Hepatic enhancement during PVP(HU)	63.7 ± 11.8	49.9 ± 12.8	< 0.001
Hepatic enhancement during DP (HU)	35.6 ± 7.7	38.5 ± 7.3	0.112
Hepatic ECV (%)	29.4 ± 5.6	34.8 ± 7.9	0.001
Hepatic IWR (%)	43.0 ± 12.4	18.8 ± 20.2	< 0.001
Volumetric parameters			
LV/BSA (mL/m^2^)	823.4 ± 149.2	734.1 ± 150.1	0.026
SpV/BSA (mL/m^2^)	117.3 ± 60.5	188.6 ± 126.5	0.005
^99m^Tc-GSA parameters			
HH15	0.61 ± 0.07	0.73 ± 0.10	< 0.001
LHL15	0.92 ± 0.03	0.82 ± 0.10	< 0.001

Data are mean ± standard deviation or number (%)

*ICG-R15* indocyanine green retention after 15 min, *PVP* portal venous phase, *DP* delayed phase, *ECV* extracellular volume fraction, *IWR* iodine washout-rate, *LV/BSA* liver volume indexed by body surface area, *SpV/BSA* spleen volume indexed by body surface area, *GSA* galactosyl human serum albumin, *HH15 (blood clearance index)* heart counts at 15 min divided by heart counts at 3 min, *LHL15* *(liver receptor index)* liver counts at 15 min divided by heart counts plus liver counts at 15 min

### Correlation between imaging parameters and ICG-R15

**Fig. 1:**
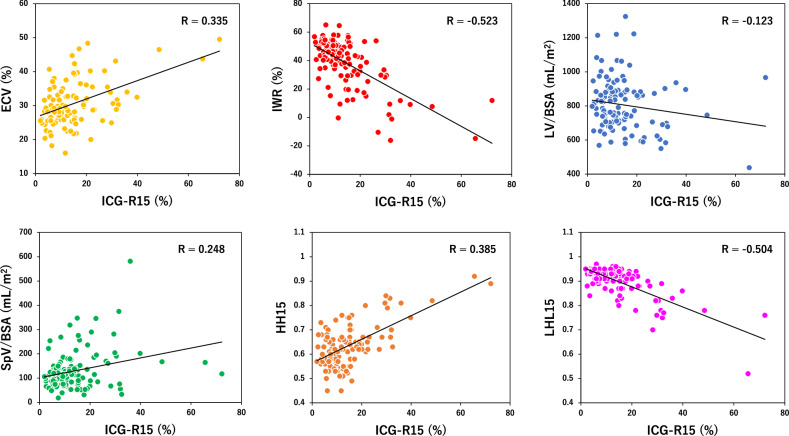
Correlations between ICG-R15 and imaging parameters. Spearman rank correlation coefficient of ECV, IWR, LV/BSA, SpV/BSA, HH15, and LHL15 were 0.355 (95% CI: 0.186–0.503), −0.523 (95% CI: −0.643 to −0.378), −0.123 (95% CI: −0.298 to 0.059), 0.248 (95% CI: 0.070–0.410), 0.385 (95% CI: 0.219–0.529), and −0.504 (95% CI: −0.628 to −0.356), respectively

There were significant negative correlations between ICG-R15 and hepatic enhancement during PVP (*r* = −0.338), IWR (*r* = −0.523) or LHL15 (*r* = −0.504), while significant positive correlations were observed between ICG-R15 and hepatic enhancement during DP (*r* = 0.243), ECV (*r* = 0.355), SpV/BSA (*r* = 0.248), and HH-15 (*r* = 0.385) (Table 3 and Fig. 1).

**Table 3:** Spearman rank correlations between ICG-R15 and imaging liver functional parameters

Parameter	*r* (95% confidence interval)
Iodine parameters	
Hepatic enhancement during PVP(HU)	−0.338 (−0.489 to −0.167)
Hepatic enhancement during DP (HU)	0.243 (0.065 to 0.406)
Hepatic ECV (%)	0.355 (0.186 to 0.503)
Hepatic IWR (%)	−0.523 (−0.643 to −0.378)
Volumetric parameters	
LV/BSA	−0.123 (−0.298 to 0.059)
SpV/BSA	0.248 (0.070 to 0.410)
^99m^Tc-GSA parameters	
HH15	0.385 (0.219 to 0.529)
LHL15	−0.504 (−0.628 to −0.356)

*ICG-R15* indocyanine green retention rate at 15 min, *PVP* portal venous phase, *DP* delayed phase, *ECV* extracellular volume fraction, *IWR* iodine washout-rate, *LV/BSA* liver volume indexed by body surface area, *SpV/BSA* spleen volume indexed by body surface area, *GSA* galactosyl human serum albumin, *HH15 (blood clearance index)* heart counts at 15 min divided by heart counts at 3 min, *LHL15 (liver receptor index)* liver counts at 15 min divided by heart counts plus liver counts at 15 min

### Diagnostic performance for predicting ICGR15 ≥ 20%

**Fig. 2:**
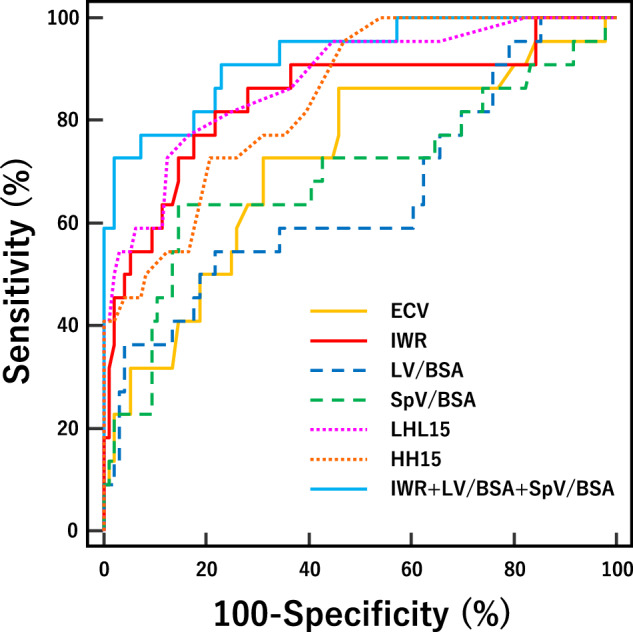
Receiver operating characteristic curves of ECV, IWR, LV/BSA, SpV/BSA, HH15, and LH15, and combined CT model (IWR + LV/BSA + SpV/BSA) to discriminate patients with ICG-R15 ≥ 20%. AUC of ECV, IWR, LV/BSA, SpV/BSA, HH15, and LHL15 were 0.719 (95% CI: 0.580–0.828), 0.845 (95% CI: 0.698–0.931), 0.653 (95% CI: 0.501–0.790), 0.694 (95% CI: 0.553–0.820), 0.844 (95% CI: 0.747–0.915), and 0.878 (95% CI: 0.759–0.944), respectively. The combination of IWR with hepatosplenic indices yielded an AUC of 0.924 (95% CI: 0.860–0.965)

The results of the ROC analysis are shown in Fig. 2 and Table 4. Among CT parameters, the highest AUC for predicting ICG-R15 ≥ 20% was achieved with IWR (0.845), followed by ECV (0.719), SpV/BSA (0.694), and LV/BSA (0.653). HH15 and LHL15 yielded AUCs of 0.844 and 0.878, respectively. LHL15 provided significantly higher AUCs than ECV (*p* = 0.009), LV/BSA (*p* = 0.007), and SpV/BSA (*p* = 0.033), while no significant difference from IWR was observed (*p* = 0.446). The multivariable analysis identified IWR, LV/BSA, and SpV/BSA as independent predictors for ICG-R15 ≥ 20%, with odds ratios of 0.894 (95% CI: 0.846–0.945), 0.986 (95% CI: 0.979–0.994), and 1.012 (95% CI: 1.003–1.021), respectively. The formula for the diagnostic model incorporating these variables is as follows:

$$P=\frac{1}{{1+e}^{-(11.869-0.1119\times {{{\rm{IWR}}}}+0.0118\times {{{\rm{SpV}}}}/{{{\rm{BSA}}}}-0.0139\times {{{\rm{LV}}}}/{{{\rm{BSA}}}})}}$$

**Table 4:** Diagnostic performance of imaging parameter for predicting ICG-R15 ≥ 20%

Parameters	Thresholds	Sensitivity	Specificity	AUC
Iodine parameters				
Hepatic ECV	≥ 30.2%	72.7% [49.8%, 89.3%]	68.8% [58.5%, 77.8%]	0.719 [0.580, 0.828]
Hepatic IWR	≤ 35.9%	81.8% [59.7%, 94.8%]	78.1% [68.5%, 85.9%]	0.845 [0.698, 0.931]
Volumetric parameters				
LV/BSA	≤ 705.8 mL/m^2^	54.6% [32.2%, 75.6%]	78.1 [68.5%, 85.9%]	0.653 [0.501, 0.790]
SpV/BSA	> 156.8 mL/m^2^	63.6% [40.7%, 82.8%]	85.4% [76.7%, 91.8%]	0.694 [0.553, 0.820]
^99m^Tc-GSA parameters				
HH15	> 0.66	72.7% [49.8%, 89.3%]	79.2% [69.7%, 86.8%]	0.844 [0.747, 0.915]
LHL15	≤ 0.89	77.3% [54.6%, 92.2%]	83.3% [74.4%, 90.2%]	0.878 [0.759, 0.944]
Combined CT model^*^				
IWR, LV/BSA, and SpV/BSA	> 0.472	72.7% [49.8%, 89.3%]	97.9% [92.7%, 99.7%]	0.924 [0.860, 0.965]

Data in brackets are 95% confidence interval

^*^ Diagnostic model incorporating IWR, LV/BSA, and SpV/BSA determined as independent predictors in multivariable analysis

*AUC* area under the receiver operating characteristic curve, *ECV* extracellular volume fraction, *IWR* iodine washout rate, *LV/BSA* liver volume indexed by body surface area, *SpV/BSA* spleen volume indexed by body surface area, *GSA* galactosyl human serum albumin, *HH15 (blood clearance index)* heart counts at 15 min divided by heart counts at 3 min, *LHL15 (liver receptor index)* liver counts at 15 min divided by heart counts plus liver counts at 15 min

**Fig. 3:**
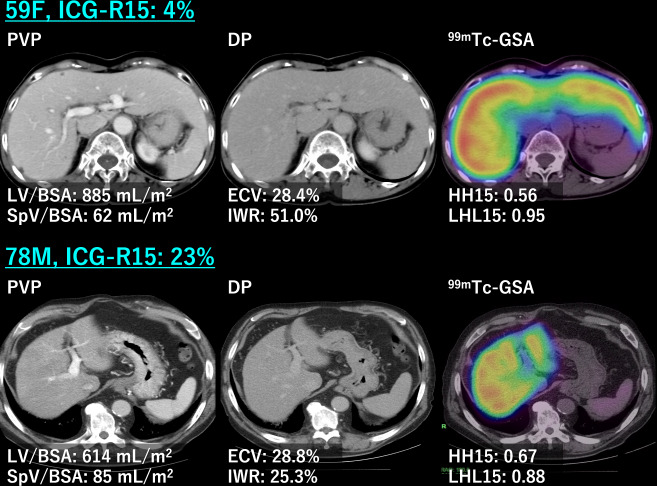
Axial contrast-enhanced CT images during PVP (left column) and DP (middle column) and fused ^99m^Tc-GSA SPECT/CT images (right column). The upper row shows a 59-year-old woman with normal liver function according to ICG-R15 (4%) and ^99m^Tc-GSA. The lower row shows a 78-year-old man with severe liver dysfunction according to ICG-R15 (23%) and ^99m^Tc-GSA. LV/BSA was higher and SpV/BSA was lower in the high-functioning patient. ECV did not reflect any difference between the patients. The IWR reflected ICG-R15 and ^99m^Tc-GSA well, being 51% in the normal functioning patient and 25% in the poor

The diagnostic model yielded the highest AUC of 0.924. The optimal threshold determined by the Youden index is > 0.472, with a sensitivity of 72.7% and a specificity of 97.9%. Representative cases are shown in Fig. 3.

## Discussion

Despite the widespread use of multiphase CT in patients with hepatobiliary diseases, its potential role in assessing liver function has been scarcely evaluated. This study demonstrated that CT parameters of ECV, IWR, LV/BSA, and SpV/BSA significantly correlate with liver function measured by ICG-R15. Among these, IWR showed the highest discriminative value for identifying patients with severe liver dysfunction (ICG-R15 ≥ 20%). Additionally, IWR, LV/BSA, and SpV/BSA were independent predictors of ICG-R15 ≥ 20%, with a combined model demonstrating improved accuracy. These findings suggest that hemodynamic and volumetric indices from multiphase hepatic CT can play complementary roles in estimating liver function.

Hepatic ECV increases as fibrosis progresses, which can be estimated with equilibrium CT [38–40]. CT-ECV has been shown as a liver fibrosis indicator with varied predictive values [41, 47–54]. To date, no studies have evaluated its diagnostic role in assessing liver function, and this is the first study to elucidate that ECV showed a weak negative correlation with ICG-R15 (*r* = 0.335) and moderate diagnostic performance for identifying patients with an ICG ≥ 20% (AUC: 0.719). ECV is essentially a static parameter reflecting hepatic enhancement at a single time point and does not fully incorporate hemodynamic changes associated with liver function, which might contribute to the limited predictive values in this study.

IWR has been recently introduced for predicting liver fibrosis severity on routine multiphase CT [41]. This study demonstrated that it showed moderate correlations with ICG-R15 (*r* = −0.523). Additionally, patients with ICG-R15 ≥ 20% exhibited substantially lower IWR values compared to those with ICG-R15 < 20%, with an AUC of 0.845 for identifying ICG-R15 ≥ 20%. Progress in portal hypertension and fibrosis deposition, associated with worsened liver function, can lead to reduced and delayed contrast inflow in the liver, as well as delayed contrast outflow due to prolonged diffusion between intravascular and expanded extracellular spaces [55–63]. IWR may reflect such hemodynamic changes on multiphase CT. Since ICG-R15 also depends on the severity of fibrosis and liver blood flow [64, 65], the observed correlation between IWR and ICG-R15 appears reasonable. Although the ^99m^Tc-GSA parameter of LHL15 allows direct estimation of functioning hepatic cells, it does not adequately capture the hemodynamic changes related to portal hypertension and fibrosis. Consequently, its correlation with ICG-R15 may remain at a level that does not show a significant difference from that of IWR.

Liver and spleen volume have been reported to correlate negatively and positively, respectively, with ICG-R15 [13, 28, 34, 35], which was confirmed in this study. Multivariable analysis showed that both LV/BSA and SpV/BSA were independent predictors for identifying patients with ICG ≥ 20%. Although each parameter had limited predictive value when used independently (AUCs of 0.653 and 0.694, respectively), combining them with IWR allowed for accurate identification of patients with ICG-R15 ≥ 20% (AUC of 0.926). This suggests that assessing hemodynamic and volumetric changes has complementary diagnostic roles. Our findings have important clinical implications, as liver function can be quantitatively predicted by routine CT performed for liver tumor assessments, potentially serving as an alternative or complement to established liver function tests.

Contrast-enhanced MRI with gadoxetic acid is another imaging technique for estimating liver function [13, 14, 66–69]. Gadoxetic acid is taken up by hepatocytes via organic anion transporters, correlating its uptake quantitative parameters with liver functional metrics. However, liver signal intensity on T1-weighted images exhibits a non-linear relationship with contrast concentration and is influenced by extracellular space contrast enhancement. The use of T1 mapping with a dual-compartment model may provide a more accurate estimation of liver function [23, 28, 67], though this method is technically demanding and not widely accessible. As a simpler alternative approach, the functional liver imaging score (FLIS), which is based on visual liver enhancement, biliary excretion, and portal vein signal intensity at the hepatobiliary phase, has been reported to predict the prognosis of chronic liver disease [70–72]. Recent studies have demonstrated that FLIS shows negative correlations with ICG-R15 (*r* = −0.258 to −0.587) [73, 74] and can predict severe liver dysfunction (ICG-R15 > 20%) with an AUC of 0.756 [74]. These findings were derived from patient populations with differing backgrounds and selection criteria, making it difficult to directly compare them with our results. Given the broader availability of multiphase hepatic CT, comparing its predictive performance with various gadoxetic acid-enhanced MRI approaches under controlled, comparable conditions could be an intriguing area for future research.

The incidence of clinically significant PHLF in our study (2/72 [2.8%]) was similar to or slightly lower than those reported in large-scale studies, including one with 949 patients (36/949 [3.7%]) [75] and another with 12,055 patients (312/12,055 [2.6%]) [76]. The relatively low incidence of clinically significant PHLF in this study may reflect the low prevalence of cirrhotic (F4) cases (15/72 [20.8%]) and rigorous surgical planning based on comprehensive preoperative liver function assessments with ICG-R15, ^99m^Tc-GSA, and CT volumetry. Given that the morbidity of severe surgical complications is influenced by various factors beyond liver function, and several investigations have shown limited associations between preoperative liver function and the severity of liver-non-specific surgical complications [77, 78], further investigation is needed to determine whether the multiphase CT approach could contribute to improved clinical outcomes.

This study has several limitations. First, it was a relatively small, single-institution study, raising concerns about inherent selection bias. External validation at multiple institutions is warranted to ensure the generalizability and robustness of our findings. Secondly, the study results were derived from our routine SPECT/CT protocol; thus, reproducibility with different contrast or scanning protocols remains to be elucidated. Thirdly, this study included a variety of liver diseases, which may have influenced the results. Fourth, a fixed tube current protocol with an older scanner was used for multiphase hepatic CT. The use of automated tube current modulation is recommended to optimize radiation dose. Fifth, ^99m^Tc-GSA is not widely available in Western countries; however, its limited accessibility motivated us to explore whether multiphase CT parameters could serve as potential predictors of hepatic functional reserve. Finally, this study did not focus on the predictive values for surgical complications, similar to numerous studies on imaging-based liver function assessments [13, 14, 67–69]. Predicting surgical complications represents a distinct study question that is influenced by numerous factors beyond liver function. As this is the first study to investigate the potential role of multiphase CT in estimating liver function within a heterogeneous patient sample, future multi-institutional studies with larger sample sizes and careful control of confounding factors are needed to analyze clinical outcomes. Since iodine uptake and volumetric parameters can be evaluated using existing CT images, our findings are expected to accelerate such future investigations.

In conclusion, IWR showed a better correlation and prediction of liver function as measured by ICG-R15 than ECV and hepatosplenic volumetry on routine multiphase hepatic CT. Combining IWR with liver and spleen volume indices demonstrated high predictive performance for identifying patients with severe liver dysfunction (ICG-R15 ≥ 20%), similar to ^99m^Tc-GSA, highlighting its promising role in estimating liver function. Further investigations are needed to establish whether this approach can predict or improve clinical outcomes.

## Abbreviations

^99m^Tc-GSA: Technetium-99m diethylenetriamine-pentaacetic acid galactosyl human serum albumin
DP: Delayed phase
ECV: Extracellular volume fraction
HH15: Blood clearance index
ICG: Indocyanine green
ICG-R15: Indocyanine green retention at 15 min
IWR: Iodine washout rate
LHL15: Liver receptor index
LV/BSA: Liver volume indexed by body surface area
MELD: Model for end-stage liver disease
SpV/BSA: Spleen volume indexed by body surface area
